# Advances in cell death mechanisms involved in viral myocarditis

**DOI:** 10.3389/fcvm.2022.968752

**Published:** 2022-08-09

**Authors:** Yang Yang, Wang Li, Benshuai You, Chenglin Zhou

**Affiliations:** ^1^Taizhou People’s Hospital Affiliated to Nanjing Medical University, Taizhou, China; ^2^Clinical Laboratory Center, Jiangsu Taizhou People’s Hospital, Taizhou, China; ^3^School of Medicine, Jiangsu University, Zhenjiang, China

**Keywords:** VMC, CVB3, apoptosis, autophagy, pyroptosis, ferroptosis, necrosis, SARS-CoV-2

## Abstract

Viral myocarditis is an acute inflammatory disease of the myocardium. Although many etiopathogenic factors exist, coxsackievirus B3 is a the leading cause of viral myocarditis. Abnormal cardiomyocyte death is the underlying problem for most cardiovascular diseases and fatalities. Various types of cell death occur and are regulated to varying degrees. In this review, we discuss the different cell death mechanisms in viral myocarditis and the potential interactions between them. We also explore the role and mechanism of cardiomyocyte death with severe acute respiratory syndrome coronavirus 2 (SARS-CoV-2) infection. Exploring the mechanisms may help in the early identification and the development of effective treatments, thus improving the quality of life of patients with viral myocarditis. We believe that the inhibition of cardiomyocyte death has immense therapeutic potential in increasing the longevity and health of the heart.

## Introduction

Myocarditis is the inflammation and injury of the myocardium and can be caused by several viruses. However, coxsackievirus B3 (CVB3) is still considered the most common etiological agent of viral myocarditis (VMC). In recent years, the novel severe acute respiratory syndrome coronavirus 2 (SARS-CoV-2) has also been detected in the heart muscle of infected patients during the outbreak of coronavirus disease 2019 (COVID-19) (1). VMC is a serious immune-mediated clinical condition that is characterized by excessive inflammatory lesions of the myocardium (2, 3). The observed pathology in VMC results from interactions between viral processes and host immune responses at various stages of disease, leading to non-specific myocardial interstitial inflammatory lesions (4). Both innate and adaptive immune responses are crucial determinants of the severity of myocardial damage and contribute to the development of chronic myocarditis and dilated cardiomyopathy following acute VMC (5).

Viral myocarditis incidence has been increasing in developing countries, especially China. According to the available clinical evidence, VMC-associated mortality in young people is as high as 21%, and sudden deaths due to VMC or fatal ventricular arrhythmias in children account for approximately 20% of deaths (6). Animal models of VMC predict a maladaptive postviral immune-mediated response, which leads to eventual myocardial cell dysfunction and compromised contractility (7). Patients with persistent viral infections in the myocardium are likely to develop dilated cardiomyopathy and congestive heart failure (8).

Cell death is a key component of the host defense against microbial infection, which is critical to maintaining tissue homeostasis and essential biological functions; changes in this process have significant pathological implications. Cell death includes apoptosis, autophagy, pyroptosis, ferroptosis, and necrosis. VMC caused by CVB3 and SARS-CoV-2 is associated with cell death. For CVB3 and SARS-CoV-2, NOD-, LRR- and pyrin domain-containing protein 3 (NLRP3), in concert with myeloid differentiation factor-88 (MyD88), drastically increased the production of pro-inflammatory (9). NLRP3 inflammasome being upstream of cytokine storm in CVB3 or SARS-CoV-2 caused VMC has been reported to be a considerable therapeutic target (10). In addition, it has revealed that MyD88 is a key contributor to cardiac inflammation, mediating cytokine production (11). MyD88 inhibitors could also be useful for myocarditis therapy. Here, we systematically review the cell death mechanism of VMC and speculate on the potential therapeutic options.

## Viral myocarditis and apoptosis

Apoptosis is programmed cell death that is characterized by the formation of apoptotic bodies. To initiate apoptosis, cells activate caspase-3 through exogenous death receptors and endogenous mitochondrial pathways. Apoptosis is required for homeostasis maintenance and is involved in many pathophysiological processes, including ischemia, hypoxic injury, and viral pathogenesis (12, 13). Although no consensus exists on the specific pathogenesis of VMC, myocardial apoptosis plays an indispensable role in VMC pathogenesis (14). In 1994, Kawano et al. (15) first reported the presence of multifocal cardiomyocyte apoptosis by analyzing myocardial biopsy samples of patients with chronic myocarditis. Henke et al. (16) and Kyto et al. (17) have observed numerous apoptotic cardiomyocytes and a significant increase in caspase-3 activity in mice infected with CVB3, indicating that cardiomyocyte apoptosis is involved in the pathogenesis of myocarditis. Apoptosis is also related to VMC-induced heart damage and leads to myocardial remodeling (18). Thus, apoptosis may play a vital role in cardiomyocyte death in VMC and is associated with the development of fatal heart failure (5).

**FIGURE 1:**
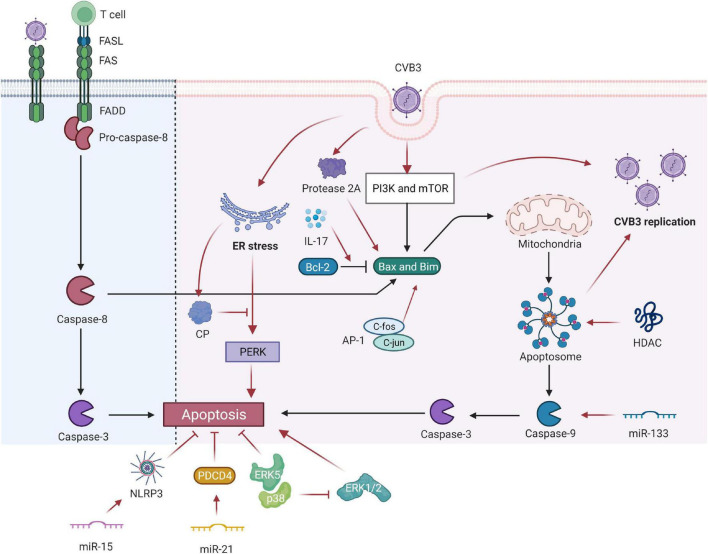
Coxsackievirus B3 (CVB3) manipulates cell apoptosis. CVB3 infection can markedly induce myocardial apoptosis via death receptor–mediated and mitochondrial-mediated signaling pathways. Viral infection induces abnormal expression of Fas antigen in the myocardium and cross-links with FasL of active cells. Activated CTL binds to target cells via Fas/FasL and causes apoptosis. The PI3K and mTOR-signaling pathways participate in the CVB3-induced VMC by mediating the proapoptosis factor Bim, Bax, caspase-9, caspase-3, and viral replication. The inhibition of HDAC activity increases CVB3 replication by enhancing autophagosome formation and ensuing increased myocardial apoptosis. IL-17A mediated cardiomyocyte apoptosis by regulating the Bax/Bcl-2 ratio. miR-133 targets caspase-9 and promotes cardiac cell apoptosis. miR-15 could suppress cell viability and promote CVB3-induced cell apoptosis by modulating the NLRX1-mediated NLRP3 inflammasome. miR-21 alleviates CVB3-induced myocarditis by protecting myocardial apoptosis and repressing PDCD4 expression. CVB3-stimulated cytotoxicity can be inhibited by kinase ERK5, coupled with p38 kinase activity. However, p38 promotes apoptosis through ERK1/2 inhibition indirectly. c-Fos can compose AP-1 with c-jun gene products that regulate the transcription of apoptosis-related genes. Picornavirus protease 2A in CVB3 induced apoptosis through multiple converging pathways. ER-initiated apoptosis was induced by CVB3-infected cardiomyocytes and caused myocardial apoptosis through ER stress by the PERK pathway. CP, which is located within the endoplasmic reticulum Ca2+ binding proteins, can relieve ERS-initiated apoptosis in VCM. CVB3, coxsackievirus B3; CTL, cytotoxic T lymphocytes; ER, endoplasmic reticulum; Bcl-2, B-cell lymphoma 2; Bax, Bcl-2-associated X protein; HDAC, histone deacetylase; NLRP3, NLR family pyrin domain containing 3; PDCD4, programmed cell death 4; ER, endoplasmic reticulum; CP, calumenin protein.

Coxsackievirus B3 infection often markedly induces myocardial apoptosis. The inhibition of histone deacetylase (HDAC) activity increases CVB3 replication by enhancing autophagosome formation and ensuring increased myocardial apoptosis, resulting in aggravated VMC (2). CVB3 infection in the heart activates cardiomyocyte apoptosis in both mice and humans (19). Viral invasion of myocardial cells evokes many host cell responses, such as persistent chronic inflammation, subsequently resulting in myocardial cell hypertrophy, myocardial apoptosis, and myocardial fibrosis, causing tissue damage and virus dissemination through incompletely characterized host cell signaling networks. Identifying and suppressing the mechanisms of CVB3-mediated cardiomyocyte apoptosis are critical (Figure 1).

Coxsackievirus B3 infection can markedly induce myocardial apoptosis through death receptor-mediated and mitochondrial-mediated signaling pathways, and apoptosis is also often evidenced in patients with acute myocarditis (20). A model analysis revealed that CVB3-stimulated cytotoxicity was inhibited by kinase ERK5 coupled with p38 kinase activity. By contrast, p38 indirectly promotes apoptosis through ERK1/2 inhibition but directly causes CVB3-induced necrosis (21). The Fas antigen, an important protein product of the apoptosis-promoting gene, initiates the cell death pathway by interacting with its natural ligand FasL, eventually leading to the apoptosis of target cells with positive expression of Fas (22). Viral infection induces the abnormal expression of the Fas antigen in the myocardium, and the antigen cross-links with FasL of active cells. When activated cytotoxic T lymphocytes (CTLs) bind to target cells through Fas/FasL, apoptotic signals can be transferred to the latter, leading to the apoptosis of target cells within several hours. Apoptosis induced by the Fas/FasL system is associated with the development and progression of VMC. Shenqi Fuzheng injection relieves VMC by downregulating Fas and FasL protein expression and inhibiting cell apoptosis (23).

Activation of proapoptotic mediators may be another mechanism of CVB3-induced apoptosis (24). PI3K and mTOR-signaling pathways participate in CVB3-induced VMC by mediating the proapoptosis factor Bim, Bax, caspase-9, caspase-3, and viral replication (25). In myocardial I/R injury, IL-17A induces cardiomyocyte apoptosis and neutrophil infiltration. An *in vitro* study concluded that IL-17A mediated cardiomyocyte apoptosis by regulating the Bax/Bcl-2 ratio (3). miRNA can decrease cardiomyocyte apoptosis by mediating the expression of apoptosis-related genes in the hearts of VMC mice (26). miR-133 targets caspase-9 and promotes cardiac cell apoptosis by interfering with the expression of mRNA (27). Tong et al. reported that miR-15 dysregulation is closely associated with VMC. They also demonstrated that miR-15 can suppress cell viability and promote CVB3-induced apoptosis, and its inhibition protects against CVB3-induced myocardial cell injury by modulating NLRX1-mediated NLRP3 inflammasomes (28). miR-21 can alleviate CVB3-induced myocarditis by protecting myocardial apoptosis by repressing programmed cell death 4 (PDCD4) expression (29).

In addition, c-fos plays a role in inducing apoptosis (30). Abnormal expression of c-fos may play a role in inflammatory diseases such as VMC. Its expression is increased in the cardiomyocytes of VMC mice. However, c-fos can compose AP-1 with c-jun gene products that modulate the transcription of apoptosis-related genes, thereby indirectly regulating cardiomyocyte apoptosis in VMC (31). Thus, c-fos plays a vital role in myocardial lesions and is likely to be involved in VMC pathogenesis (32).

The endoplasmic reticulum stress (ERS) reaction was discovered to be a signal transduction pathway mediating apoptosis. ER-initiated apoptosis was induced by CVB3-infected cardiomyocytes and caused myocardial apoptosis through ER stress via the PERK pathway. Calumenin protein (CP), located within the endoplasmic reticulum Ca^2+^ binding proteins, can relieve ERS-initiated apoptosis in VCM (33).

Coxsackievirus B3 can also produce viral proteins, such as protease 2A, that cause direct myocardial injury. Protease 2A inhibits host cell protein synthesis, cleaves host protein dystrophin, and may induce cardiomyopathy (34–36) and cardiomyocyte apoptosis. By transfecting individual protease genes of CVB3 into HeLa cells, Chau et al. demonstrated that protease 2A induced apoptosis through multiple converging pathways that activate proapoptotic mediators and inhibit translation and transcription (24).

## Viral myocarditis and autophagy

Autophagy, or cellular self-digestion, is a significant cellular catabolic pathway, especially for the degradation of proteins and organelles by a lysosomal pathway for maintaining cytoplasmic homeostasis (37). It plays a key role in cellular defense by removing intracellular pathogens, such as viruses, bacteria, and parasites (38). It was initially considered a primary cell survival mechanism for supplying nutrients and energy to prolong cell survival under stress conditions (38, 39). It is now associated with human disease and physiology (40). Autophagy primarily protects organisms against diverse pathologies, including infection, cancer, neurodegeneration, aging, and heart diseases (41). Thus, it is a critical cellular event associated with VMC.

**FIGURE 2:**
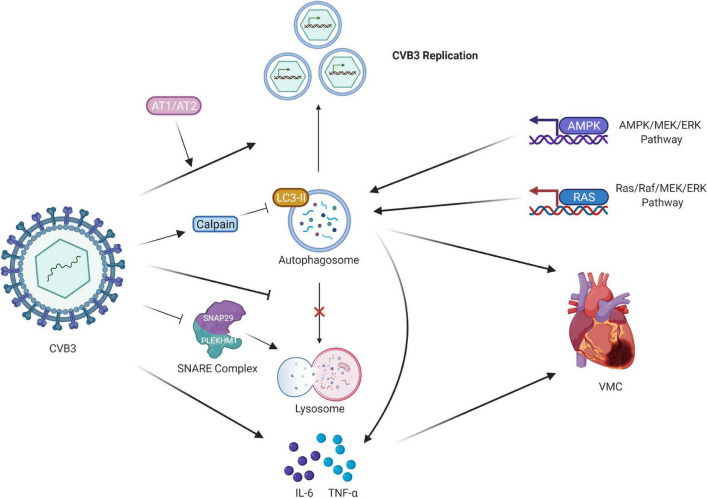
Regulation of CVB3 and autophagy in VMC. CVB3 infection triggers the formation of autophagosomes and uses the autophagosomal pathway for replication. CVB3 infection in cardiomyocytes activated calpain and increased. The inhibition of calpain activity led to the accumulation of LC3-II protein expression, impairing the autophagic flux. AT1 and AT2 regulate cardiomyocyte autophagy activity by propagating viral replication, thus triggering autophagic cell death. CVB3 might directly or indirectly induce autophagy via the AMPK/MEK/ERK and Ras/Raf/MEK/ERK signaling pathways. CVB3 inhibits the fusion of lysosomes with autophagosomes. CVB3 specifically targets the SNARE protein SNAP29 and the adaptor protein PLEKHM1, both of which regulate autophagosome fusion, for cleavage through the catalytic activity of viral proteinase 3C; this process ultimately impairs the formation of SNARE complexes. The release of proinflammatory cytokines also participates in the cardiac fibroblasts caused by CVB3 infection, and the downregulation of autophagy suppresses them. CVB3, coxsackievirus B3; VMC, viral myocarditis; LC3, light chain 3; AT1 and AT2, Type I and II angiotensin II receptors.

Autophagy prevents many infections by inducing lysosomal-mediated degradation of invading pathogens. However, previous *in vitro* studies have suggested that some enteroviruses not only evade these protective effects but also exploit autophagy to facilitate their replication (42). Various viruses can stimulate the autophagic response to elevate their replication (43). Autophagy is a “double-edged sword” for CVB3. On the one hand, autophagy can clear a small portion of CVB3 (44). However, the life cycle of CVB3 depends on autophagy (45). CVB3 infection triggers the formation of autophagosomes without promoting protein degradation by the lysosome. However, enhanced autophagosomes acting as viral RNA replication sites are exploited by CVB3 to facilitate viral replication, leading to myocardial apoptosis (44, 46). The autophagic response was induced by CVB3 infection in mouse cardiac myocytes (47). Additionally, the interplay between CVB3 and autophagy has been verified in an *in vivo* study. The cell apoptosis rate of myocardial cell H9c2 was enhanced after CVB3 infection, indicating reduced cell survival ability (48). These data highlight the major impact of autophagy on CVB3 infection (Figure 2).

Types I and II angiotensin II receptors (AT1 and AT2, respectively) regulate cardiomyocyte autophagy activity (49). AT1 triggers autophagy in neonatal cardiomyocytes as well as subsequent autophagic cell death, and AT2 counteracts the process. These alterations may have a contrary effect on virus-infected cardiomyocytes, as they propagate viral replication, thus triggering autophagic cell death. CVB3 might directly or indirectly induce autophagy in host cells through the AMPK/MEK/ERK and Ras/Raf/MEK/ERK signaling pathways, representing a key mechanism by which CVB3 regulates the number of autophagosomes (50).

In addition to the formation of autophagosomes, the release of proinflammatory cytokines participates in the activation of cardiac fibroblasts caused by CVB3 infection. The cardiac fibroblasts not only support viral replication but also participate in inflammation responses by expressing proinflammatory cytokines. A significantly increased assembly of autophagosomes was found in cardiac fibroblasts both *in vitro* and *in vivo* after CVB3 infection. CVB3 infection increased the release of proinflammatory cytokines, IL-6 and TNF-α, and autophagy downregulation suppressed their expression in cardiac fibroblasts (51).

Another mechanism is that CVB3 hijacks the autophagic machinery to facilitate its own propagation. A series of *in vivo* and *in vitro* experiments have demonstrated that CVB3 inhibits autophagic flux by significantly limiting the fusion of autophagosomes with lysosomes and/or late endosomes. Furthermore, the loss of SNAP29/PLEKHM1 inhibits autophagic flux, resulting in increased viral replication. CVB3 specifically targets the SNARE protein SNAP29 and the adaptor protein PLEKHM1, both of which regulate autophagosome fusion, for cleavage through the catalytic activity of viral proteinase 3C, ultimately impairing the formation of SNARE complexes (52). Microtubule-associated protein light chain 3 (LC3) existing in autophagosomes is essential for autophagosome formation and serves as an autophagosome marker (53, 54). Calpains are calcium-activated neutral cysteine proteases. CVB3 infection in cardiomyocytes activates calpain and increases the calpain substrate spectrin fragment. The inhibition of calpain activity both *in vitro* and *in vitro* led to LC3-II protein accumulation, impairing the autophagic flux, which may have increased viral replication and exacerbated VMC symptoms in mice due to myocardial inflammation and cardiac dysfunction. This may subsequently reduce virus autolysosome degradation (55).

## Viral myocarditis and pyroptosis

Pyroptosis is a unique inflammatory form of programmed cell death. It involves gasdermin family–mediated membrane pore formation and subsequent cell lysis, followed by the secretion of a number of proinflammatory cytokines, mainly IL-1β, IL-18, and HMGB1 (56, 57). Pyroptosis is involved in several cardiovascular diseases, including atherosclerosis, myocardial infarction, diabetic cardiomyopathy, and reperfusion injury. It is also associated with the pathogenesis of myocarditis (58). Its morphological characteristics, occurrence, and regulatory mechanisms differ from those of apoptosis and necrosis (59). It is likely initiated by the canonical caspase-1-dependent and non-canonical caspase-4/5/11-mediated (human caspase-4/5 and murine caspase-11) pyroptosis pathways (60).

Pyroptosis most frequently occurs during the infection of intracellular pathogens and may be a part of the defense mechanisms of the host against infection (56). In this process, cells recognize intracellular pathogens through many pattern-recognition receptors (PRRs) and form a multiprotein complex—the inflammasome—which activates caspase-1 (56). Gasdermin D (GSDMD) is the executioner of proptosis, which is the substrate of proinflammatory caspases (caspase-1, –11, –4, and –5). The cleaved GSDMD forms non-selective pores in the plasma membrane, leading to pyroptosis (61, 62). Activated caspase-1 converts pro-IL-1β and pro-IL-18 to their mature forms (63). The secretion of IL-1β and IL-18 and the release of cellular content due to cell lysis promote the recruitment of inflammatory cells, resulting in the activation of immune cells and the further production of cytokines and subsequently causing pathological consequences (64).

**FIGURE 3:**
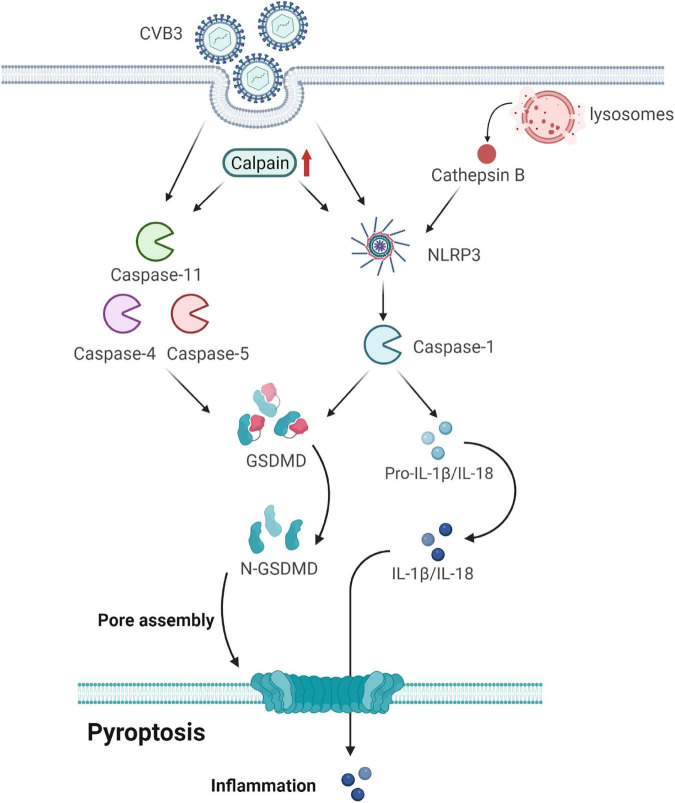
Gasdermin D (GSDMD) forms membrane pores to cause pyroptosis. CVB3 infection initiates pyroptosis by canonical caspase-1-dependent and non-canonical caspase-4/5/11-mediated pyroptosis pathways. In the canonical pyroptosis pathway, cells recognize intracellular pathogens through many PRRs and form NLRP3, which activates caspase-1. Caspase-1 processes and activates IL-1β and IL-18; it also cleaves GSDMD to release the membrane pore-forming GSDMD-N domain. GSDMD-N pores promote the release of activated IL-1β and IL-18. In the non-canonical pyroptosis pathway, cytosolic LPS binds to caspase-4/5/11 and triggers the cleavage of GSDMD but not of IL-1β and IL-18. Calpain is activated after CVB3 infection, accompanied by an increase in pyroptosis by promoting the canonical NLRP3 inflammasome/caspase-1-mediated and non-canonical caspase-11-mediated pyroptosis pathways. CVB3 infection damages the lysosomes and releases the lysosomal contents, including cathepsin B, into the cytosol. Cathepsin B exaggerates VMC by regulating the activation of NLRP3. GSDMD, gasdermin D; CVB3, coxsackievirus B3; PRRs, pattern-recognition receptors; LPS, lipopolysaccharide; VMC, viral myocarditis.

Coxsackievirus B3 infection can activate pyroptosis. Wang et al. first reported that the activation of NLRP3 inflammasome was involved in CVB3-induced myocarditis (65). Caspase-1 activation and the increased expression of IL-18 and NLRP3 were demonstrated in HeLa cells infected with CVB3 (66). The suppressed pyroptosis alleviated the inflammatory response of the virus-infected mice and reduced the replication of viruses in the myocardium, suggesting that pyroptosis is involved in the pathogenesis of CVB3 infections (Figure 3).

Calpain is activated in CVB3-infected mouse hearts, accompanied by an increase in pyroptosis. It increases apoptotic myocardial cell death and interferon (IFN)-γ and IL-17 production in the local myocardium of VMC mice (67). Moreover, it drives pyroptotic vimentin cleavage, intermediate filament loss, and cell rupture during pyroptosis (68). Its inhibition attenuates VMC by suppressing the canonical NLRP3 inflammasome/caspase-1-mediated and non-canonical caspase-11-mediated pyroptosis pathways (69).

Cathepsin B (CatB), an intracellular cysteine proteolytic enzyme, is widely expressed in various cells and is located mainly in the lysosomes. It is involved in viral infectious diseases because of its relations with viral entry and replication as well as virus-mediated cell apoptosis and immune responses (70). It aggravates CVB3-induced VMC probably by activating the inflammasome and promoting pyroptosis (58, 71). Specifically, CVB3 infection can damage lysosomes and release lysosomal contents, including CatB, into the cytosol. The activated caspase-1 then cleaves gasdermin D, releasing its N-terminal domain, which oligomerizes in the membranes to form large pores, causing membrane rupture and cell death (72). Thus, CatB may exaggerate VMC by regulating inflammasome activation.

## Viral myocarditis and necrosis

Necrosis was considered as an unregulated form of cell death, but a growing number of studies have discovered that necrosis plays a vital role in cell death and that it is regulated (59). Necroptosis is mediated by the ligands and stimuli of death receptors and executed through induction of the RIP1–RIP3 (receptor-interacting proteins 1 and 3) necroptotic complex and production of mitochondrial reactive oxygen species (ROS), followed by depletion of cellular energy under apoptotic-deficient conditions (73, 74). It is a caspase-independent necrotic cell death program regulated by receptor-interacting protein kinases, and it plays a prominent role in multiple human diseases (75).

Necrosis may be the preferred outcome for CVB3, which is counteracted by the host cell drive to die by apoptosis (21). RIP1/RIP3 was highly expressed in cardiomyocytes in the acute VMC mouse model, and downregulating its expression through the necroptosis pathway-specific blocker Nec-1 markedly alleviated myocardial damage. Thus, necroptosis plays a significant role in cardiomyocyte death and is a major pathway for cell death in acute VMC (76).

## Viral myocarditis and ferroptosis

Ferroptosis is an emerging novel form of programmed cell death, which is characterized by the production of cellular ROS from accumulated iron and lipid peroxidation (77, 78). Ferritin is the major intracellular iron storage protein complex, which includes ferritin light polypeptide 1 (FTL1) and ferritin heavy polypeptide 1 (FTH1) (79). Increased ferritin expression limits ferroptosis (80).

Acyl-coenzyme A synthetase long-chain family member 4 (ACSL4), a key component of ferroptosis, is involved in viral replication organelle formation. Enteroviruses can induce ferroptosis through ACSL4. However, its inhibitors can reduce enteroviral yields (81).

## Severe acute respiratory syndrome coronavirus 2 and viral myocarditis

Severe acute respiratory syndrome coronavirus 2, which causes COVID-19, can also cause VMC, a rare cardiovascular complication of COVID-19 (82–85). However, data on VMC caused by SARS-CoV-2 remain scarce. In addition to the direct presence of SARS-CoV-2 in the myocardium of patients with COVID-19, SARS-CoV-2 can cause myocarditis through other indirect mechanisms (86).

Similar to CVB3, SARS-CoV-2 inhibits apoptosis signaling in the initial stage of viral infection for efficient replication, with a shift to viral release in later stages; thus, enteroviruses induce the host cell toward apoptosis (87, 88). Autophagosomes are also manipulated in SARS-CoV-2 infection. Nsp3a of SARS-CoV-2 can block autophagy and accumulate autophagosomes by disrupting Rab7–HOPS complex formation to inhibit lysosome–autophagosome fusion (89).

Histopathological analysis in a patient with COVID-19 revealed active myocarditis, and the immunohistochemical marker (oxidized phosphatidylcholine) of ferroptosis was positive in the myocardial tissue, indicating its involvement in SARS-CoV-2 infection (82). Altered iron metabolism, depletion of glutathione (GSH), inactivation of glutathione peroxidase 4 (GPX4), and upregulation of PUFA peroxidation by ROS are crucial to ferroptosis; this indicates a relationship between them and the proposed mechanisms of SARS-CoV-2 infection and ferroptosis induction (90, 91).

### Dysregulation of iron metabolism in severe acute respiratory syndrome coronavirus 2 infection

Optimal iron levels within host cells are necessary for viral replication (92). VMC caused by SARS-CoV-2 can cause a cytokine storm, especially IL-6, and alter systemic iron metabolism (93, 94). The cytokine storm may promote hyperferritinemia, which can further intensify the inflammation. Moreover, elevated ferritin levels trigger nuclear receptor coactivator 4 (NCOA4)-mediated ferritinophagy. Overexpression of NCOA4 increases ferritin degradation and promotes iron release. The increased iron converts phospholipid LOOH to hydroxyl radicals through the Fenton reaction, eventually inducing ferroptosis and promoting cellular damage (90, 95–97).

### Glutathione-glutathione peroxidase 4 axis in severe acute respiratory syndrome coronavirus 2 infection

Glutathione deficiency plays a key role in SARS-CoV-2 infection (98). Moreover, the mRNA expression of ferroptosis-associated GPX4, DNA synthesis–related thioredoxin reductase, and endoplasmic reticulum-resident selenoproteins is suppressed by SARS-CoV-2 (99). Thus, both a low GSH pool and downregulation of GPX4 gene expression caused by SARS-CoV-2 infection facilitate ferroptosis (90).

### Reactive oxygen species generation during severe acute respiratory syndrome coronavirus 2 infection

**FIGURE 4:**
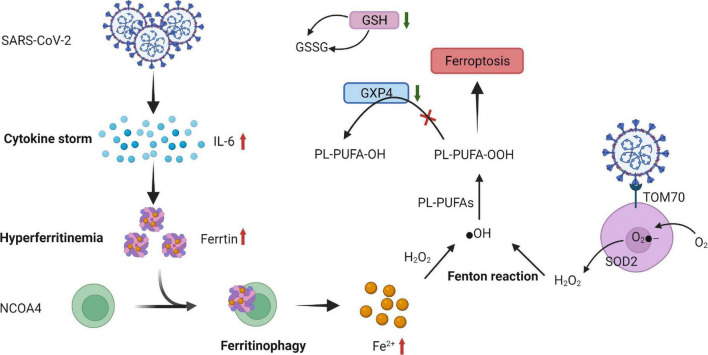
Proposed mechanism of ferroptosis in SARS-CoV-2 infection. SARS-CoV-2-related increase in cytokines, especially IL-6 causing hyperferritinemia, is characterized by the increase in intracellular iron and ferritin. This increased ferritin binds to NCOA4 and is delivered to autophagosomes, causing ferritinophagy and triggering an increase in the labile iron pool, which induces OH through the Fenton reaction and, eventually, through PL-PUFA peroxidation, which promotes ferroptosis. The expression of GSH and ferroptosis-associated GPX4 is suppressed by severe acute respiratory syndrome coronavirus 2 (SARS-CoV-2) infection. Moreover, a low GSH pool and downregulation of GPX4 gene expression caused by SARS-CoV-2 infection facilitate ferroptosis. Orf9b, one of the accessory proteins of SARS-CoV-2, increases ROS generation by binding to TOM70 at the surface of the mitochondria membrane. O_2_- is produced by ETC on the internal membrane of the mitochondria and then converted to further H_2_O_2_ by SOD and eventually, by Fenton reaction, transformed into ⋅OH, triggering LOOH generation from PUFAs that promotes ferroptosis. SARS-CoV-2, severe acute respiratory syndrome coronavirus 2; IL-6, interleukin-6; NCOA4, nuclear receptor coactivator 4; ⋅OH, hydroxyl radical; PL-PUFAs, phospholipid polyunsaturated fatty acids; PUFA-OH, phospholipid polyunsaturated fatty acid alcohols; PL-PUFA-OOH, phospholipid polyunsaturated fatty acid peroxides; GSH, glutathione; GSSG, oxidized glutathione; GPX4, glutathione peroxidase; Orf9B, open reading frame-9b; ROS, reactive oxygen species; TOM70, translocase of outer membrane 70; O_2_-, superoxide; ETC, electron transport chain; H_2_O_2_, hydrogen peroxide; SOD, superoxide dismutase; LOOH, peroxides.

Reactive oxygen species are considered to be key signals in ferroptosis (100). A study revealed that SARS-CoV-2 affects mitochondrial ROS generation through the following mechanism: one of its accessory proteins, open reading frame-9b (Orf9b), can modify mitochondrial morphology, interfere with the mitochondrial antiviral signaling system, suppress IFN production, and raise autophagy (101, 102) (Figure 4).

## Discussion

**FIGURE 5:**
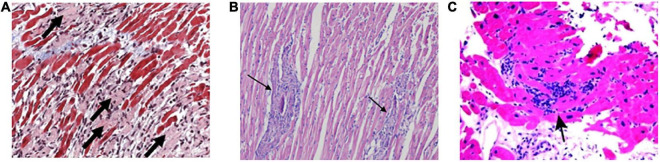
The main characteristics of histology in VMC. **(A)** Active myocarditis is characterized by an inflammatory cellular infiltrate with numerous necrotic myocytes. **(B)** Representative histopathology in a borderline myocarditis group. The inflammatory region is showed with several large foci of cellular infiltrations. **(C)** Diffuse lymphocytic infiltration of myocardium is described as lymphocytic, eosinophilic, or granulomatous in endomyocardial biopsy specimens. VMC, viral myocarditis.

Viral myocarditis typically results from infection by a cardiotropic virus, followed by active inflammatory destruction of the myocardium, which is an acute inflammatory disease of the heart, and VMC is currently a principal cause of sudden death in children and young adults (103). Some viruses like enteroviruses, adenoviruses, parvovirus B19, human herpesvirus 6, HIV, and SARS-CoV-2 were detected in patients with VMC and their biopsy specimens often showed myocarditis inflammatory (Table 1 and Figure 5) (104, 105). Nevertheless, the specific pathogenetic mechanisms underlying VMC remain unclear. Exploring these mechanisms may help in the early identification of the disease and the development of effective treatments, thus improving the quality of life of patients with VMC and reducing mortality.

**TABLE 1:** Main viruses and some microscopic characteristics of myocardial damage.

Viral	
**Classification**	**Description**

RNA viruses	Enteroviruses, HIV, SARS-CoV-2
DNA viruses	Adenoviruses, parvovirus B19, human herpesvirus 6
**Microscopic characteristics of myocardial damage**	
Active myocarditis	Inflammatory cellular infiltrate with evidence of myocyte necrosis [Figure 5A(122)]
Borderline myocarditis	Inflammatory cellular infiltrate without evidence of myocyte injury [Figure 5B (123]
Progressive inflammatory infiltrate	Lymphocytic, eosinophilic, or granulomatous [Figure 5C (124)]

Three genetically defined cell death pathways exist: apoptosis, necroptosis, and pyroptosis (106). Although research on programmed cell death initially focused on apoptosis, necrosis, pyroptosis, and other novel forms of programmed cell death such as ferroptosis have been increasingly attracting attention (107). The data showed that the expression of the autophagic activation marker LC-3II, the apoptosis marker caspase-3, and the necroptosis markers RIP1 and RIP3 was increased in the heart tissue of CVB3-infected mice. Features of all three pathways are concurrently observed in failing hearts, implying their simultaneous involvement in the pathological process of acute VMC.

The functional relationship between apoptosis and autophagy is complex. Autophagy is a prosurvival response against apoptosis. Under some conditions, autophagy can exhibit a stress adaptation that prevents cell death and suppresses apoptosis (108). The dysregulation of autophagy may decrease the viability of virus-infected cardiomyocytes because it cannot protect the host from virus-induced apoptosis. However, an increase in polyubiquitinated proteins due to the insufficient induction of autophagy in cardiomyocytes may increase endoplasmic reticulum stress and apoptosis. Apoptosis is long considered the principal process of cell death in cardiomyocytes, but programmed necrosis or necroptosis may play a vital role in cardiomyocyte cell death. Recently, caspase-8 was reported to cleave GSDMD, leading to caspase-8-mediated GSDMD-dependent cell death in response to extrinsic triggers of apoptosis (109). These examples illustrate that different forms of pyroptotic cell death exist, and that these are interconnected with apoptotic and necroptotic pathways (75).

Viruses are involved in various cell death mechanisms, including apoptosis, necroptosis, and pyroptosis (110). They activate many host cell signaling pathways, thereby evoking many host cell responses. Coxsackie B viruses (most commonly CVB3) are responsible for most VMC cases. Approximately 25% cases of dilated cardiomyopathy and myocarditis in children and young patients are caused by CVB3 (111). CVB3 is a small, non-enveloped, single-strand plus RNA enterovirus in the Picornaviridae family, and is considered the leading cause of VMC because it has the strongest myocardial affinity (25). A major component of CVB3 pathogenesis is the death of infected cardiomyocytes, which damages myocardial cells directly or indirectly through autoimmune reactions, leading to their degeneration and necrosis or interstitial inflammatory cell infiltration and fibrosis, which furthers cardiomyocyte injury and loss and, thus, myocardial dysfunction (112, 113). Throughout infection, CVB3 modulates various cell signaling pathways that enable virus propagation (114). It can trigger a direct cytopathic effect and induce apoptosis in HeLa cells and mouse hearts (115). It uses different strategies, including direct damage to host cells followed by a host inflammatory response to CVB3 infection and cell death to super-additively promote target organ tissue injury and dysfunction (113). Notably, CVB3-induced acute myocarditis is almost certainly the early effect of direct virus-induced myocyte damage, followed by host immune and inflammatory responses, the intensity of which is partly related to persistent or chronic CVB3 infection.

Several programs of cell death for SARS-CoV-2 are similar to well-known cardiogenic viruses CVB3. The incidence of SARS-CoV-2–induced myocarditis remains unknown due to insufficient data. SARS-CoV-2 appears to have analogous effects on the heart as other myocarditis-causing viruses, but further studies of the effects of SARS-CoV-2 on the heart are warranted.

In addition to the related mechanisms mentioned above, non-pharmacological strategies aimed to treat myocarditis should also be mentioned. Related studies have found that natural compounds and herbal medicines have protective effects against VMC (116). Some of the nutraceuticals, such as medicinal mushrooms, ascorbic acid, quercetin, and polydatin may play a role in the treatment of VMC (117, 118). For example, some natural molecules such as berberine, quercetin, and apigenin have been found to be effective in relieving experimental autoimmune myocarditis, which may be related to their mitigation of oxidative stress and inflammatory cytokines (119, 120). Besides, some studies have indicated that alkaloids such as berberine show benefits in myocarditis through modulating Th17 and Th1 cell differentiation (121). Although these therapies show potential, additional efforts for clinical trials are requested. In short, the combination of non-pharmacological therapy and traditional therapy may be an effective strategy for the treatment of VMC, especially during the pandemic period when the risk of myocarditis is higher in COVID-19.

## Summary and future directions

Viral myocarditis usually affects children and young adults, and its main long-term consequences are dilated cardiomyopathy and chronic heart failure. Although the pathophysiology of myocarditis has been well studied in experimental animal models, few human studies have analyzed the cellular processes contributing to myocardial damage in myocarditis. In this review, we synthesized the data on how viruses, especially CVB3 and SARS-CoV-2, manipulate several cell death pathways, causing the myocardial effects observed in VMC. The evidence indicates that these various forms of cell death are interlinked to form a network to mediate cell availability. Future studies should elucidate this association and help develop novel treatment strategies directed toward pathway-specific targets for improving the treatment outcomes of patients with viral-induced myocarditis.

## Author contributions

CZ: administrative support. WL and BY: collection and assembly of data. YY: data analysis and interpretation. All authors were conceptualized, designed, wrote the manuscript, and approved the submitted version.
